# Case Report: Neonatal lethality caused by novel compound heterozygous missense variants of *SLC6A9* gene

**DOI:** 10.3389/fped.2026.1945449

**Published:** 2026-09-25

**Authors:** Jianbin Liu, Ke Wu, Yan Ni

**Affiliations:** 1 Pediatric Rehabilitation Department, Quzhou Maternal and Child Health Care Hospital, Quzhou, Zhejiang, China; 2 Laboratory of Prenatal Diagnosis Center, Quzhou Maternal and Child Health Care Hospital, Quzhou, Zhejiang, China; 3 Women’s Health Department, Quzhou Maternal and Child Health Care Hospital, Quzhou, Zhejiang, China

**Keywords:** clubfoot, facial dysmorphism, glycine encephalopathy, neonatal lethality, *SLC6A9* gene

## Abstract

**Background:**

The homozygous or compound heterozygous missense variants in the *SLC6A9* gene (OMIM*601019) are associated with autosomal recessive glycine encephalopathy with normal serum glycine (OMIM#617301). Fewer than ten *SLC6A9* variants have been reported to date. Neonates harboring pathogenic variants in the *SLC6A9* gene exhibited a mortality rate of up to 75% during the first 7 months of life. We reported the first documented case of neonate death in China attributable to compound heterozygous missense *SLC6A9* variants.

**Methods:**

Trio-based whole-exome sequencing (trio-WES) was performed on genomic DNA (gDNA) of amniotic fluid cells and parental peripheral blood samples. Two mutant *SLC6A9* expression vectors, namely pCMV-Flag-Neo(EGFP)-*SLC6A9*-m1 and pCMV-Flag-Neo(EGFP)-*SLC6A9*-m2 (*SLC6A9*-m1/m2), and wild-type pCMV-Flag-Neo(EGFP)-*SLC6A9*-wt (*SLC6A9*-wt) mammalian expression vectors were constructed. All vectors were subsequently transformed into HEK293T cells. Assays measuring the relative expression of *SLC6A9* gene mRNA and SLC6A9 protein were performed. Concurrently, we conducted a systematic review of previously reported *SLC6A9*-related cases with accessible clinical data, encompassing relevant genetic testing findings and prenatal/postnatal clinical manifestations.

**Results:**

Trio-WES identified novel compound heterozygous missense variants c.859C > T (p.Arg287Trp) and c.1120T > C (p.Cys374Arg) in the *SLC6A9* gene (NM_201649.4). *In vitro* functional studies demonstrated that neither variant caused a reduction in the expression of mutant mRNA, but both led to a significant decrease in the expression of mutant SLC6A9 protein. After reviewing the current literature, we found that fetuses with *SLC6A9* gene variants commonly presented with increased nuchal translucency (NT), polyhydramnios and talipes equinovarus (clubfoot). Pathogenic *SLC6A9* variants exhibited complete penetrance and a relatively consistent phenotypic spectrum characterized by developmental delay, facial dysmorphism, clubfoot, and a high neonatal mortality rate. The characteristic phenotype in affected infants was characterized by elevated cerebrospinal fluid (CSF) glycine concentrations, with or without concurrent elevation in serum glycine levels.

**Conclusion:**

Preliminary evidence from *in vitro* functional studies suggested that the pathogenic mechanism of *SLC6A9*-related glycine encephalopathy may be associated with the loss-of-function (LoF) effect caused by *SLC6A9* gene variants. This study also expanded the mutational spectrum of the *SLC6A9* gene.

## Introduction

Empirical evidence indicates that 30%–40% of neonatal deaths are associated with monogenic genetic disorders (1, 2). Herein, we report an extremely rare case of neonatal lethality caused by novel compound heterozygous missense variants of *SLC6A9* gene in China. The homozygous or compound heterozygous missense variants in the *SLC6A9* gene (OMIM*601019) are associated with autosomal recessive glycine encephalopathy with normal serum glycine (OMIM#617301). Between 2003 and 2005, multiple independent studies reported findings regarding the *Slc6a9* gene knockout animal model. The *Slc6a9* knockout mouse exhibited severe growth restriction, respiratory failure and early postnatal lethality. It was not until 2016 that Majid Alfadhel et al. (3) reported the first documented human case of a homozygous *SLC6A9* gene variant associated with a distinct neurodevelopmental phenotypes, characterized by neonatal respiratory distress, global delay, hypotonia, congenital talipes equinovarus, and elevated glycine concentrations in both plasma and cerebrospinal fluid. Fewer than ten *SLC6A9* variants have been reported to date. Pathogenic *SLC6A9* variants exhibited complete penetrance and a high neonatal mortality rate. Here, we also performed a series of functional studies on these two variants, which preliminarily suggested that the pathogenic mechanism of *SLC6A9*-related glycine encephalopathy may be associated with the loss-of-function (LoF) effect induced by *SLC6A9* gene variants.

## Materials and methods

### Clinical features

A 19-year-old woman (gravida 3, para 0) sought genetic counseling due to a neonatal death. The first pregnancy terminated by medical abortion; the second pregnancy resulted in induced labor at 19 weeks due to fetal bilateral clubfoot, without fetal genetic analysis. The present pregnancy constituted her third gestation. At 18 weeks of gestation, ultrasound examination revealed bilateral choroid plexus cysts. At 19 weeks, ultrasound showed bilateral clubfoot. Follow-up ultrasounds at 20, 22, 26, and 35 weeks confirmed the persistent bilateral clubfoot. At 19 weeks, amniocentesis was performed. The chromosomal karyotype analysis and copy number variations (using low pass genome sequencing) of the amniotic fluid sample revealed no abnormalities. At 35 weeks, the fetal Doppler ultrasound scan revealed the following fetal findings: biparietal diameter: 81 mm (−1.6 SD); head circumference: 304 mm (−1.0 SD); abdominal circumference: 310 mm (0 SD); femur length: 62 mm (−1.0 SD). The estimated fetal weight (EFW) was 2,291 ± 334 g (−0.6 SD). A female infant was delivered vaginally at 39 weeks and 5 days of gestation. The birth weight was 3,150 g, the birth length was 50.0 cm, the head circumference was 31.5 cm (−1.3 SD). The APGAR scores at 1 and 5 min were 5 and 7, respectively. The newborn exhibited profound hypotonia, absence of cry, respiratory distress, recurrent apneic episodes, hypotonia, episodic rhythmic limb tremors, arthrogryposis (bilateral clubfeet, bilateral flexion contractures of the elbows, hip dislocation, and clenched fists), absence of suck reflex, and feeding difficulties. At 3 h postpartum, no abnormalities were detected on neonatal brain ultrasound; peripheral blood glycine concentration was 699.00 μmol/L (reference range: 150–700 μmol/L), and other metabolic screening tests showed no abnormalities. The newborn presented with fair and loose skin, dysmorphic facial features (low-set ears, micrognathia, depressed nasal bridge, hypertelorism, retrognathia, deep prominent philtrum, glossoptosis, high-arched palate), bilateral talipes equinovarus and overriding toes (Figure 1). Upon admission, the newborn was diagnosed with necrotizing enterocolitis, pneumonia, and anemia, and died from respiratory failure on the fourth day after birth.

**Figure 1:**
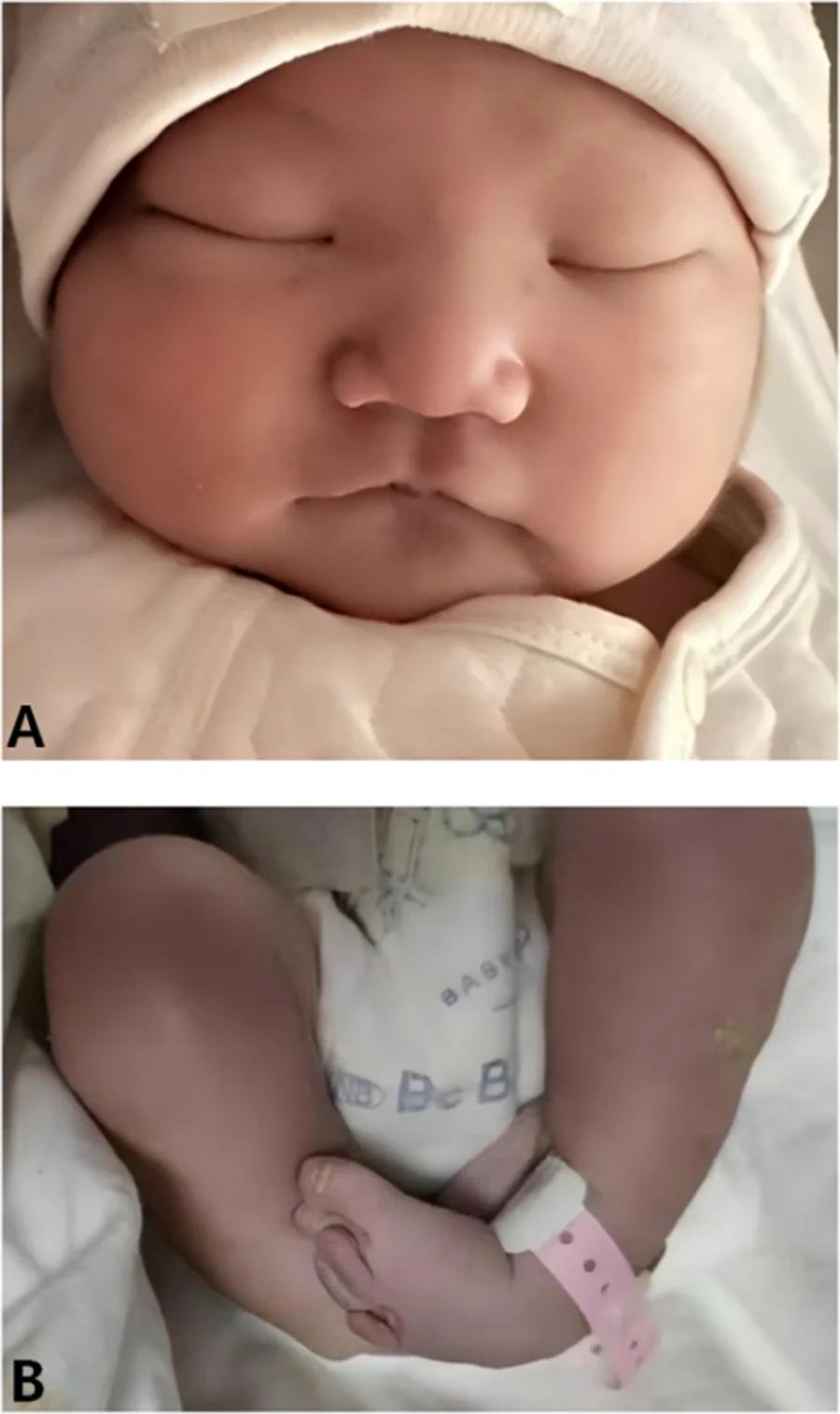
Facial appearance and limb deformities. **(A)** Distinctive facial appearance. **(B)** Bilateral talipes equinovarus and overriding toes.

### Whole-exome sequencing (WES)

Genomic DNA (gDNA) was isolated from centrifuged amniotic fluid cells using the TIANGEN Genomic DNA Extraction Kit (Tiangen, China) in accordance with the manufacturer's instructions. Parental gDNA was isolated from peripheral blood samples using Genome Column Medium Extraction Kit (Kangweishiji, China) according to the manufacturer's instructions. The extracted gDNA samples were subjected to quality control using a Qubit 2.0 fluorimeter and electrophoresis with a 0.8% agarose gel for further protocols. Trio-based whole-exome sequencing (trio-WES) was performed on fetal and parental gDNA. Quality control (QC) of the DNA library was performed using an Agilent 2100 Bioanalyzer System (Agilent, USA). DNA nanoball (DNB) preps of clinical samples were sequenced on ultra high throughput DNBSEQ-T7 platform (MGI, Shenzhen, China) with paired-end 150 nt strategy following manufacturer's protocol.

### Bioinformatic analysis

Sequencing data were analyzed according to our in-house procedures. Raw data were processed to remove adapters and filter out low-quality reads. The trimmed reads were then mapped to the University of California at Santa Cruz (UCSC) GRCh37/hg19 reference genome using Burrows-Wheeler Aligner (BWA) software (version 0.7.17). The Genome Analysis ToolKit (GATK) software (version 4.2.1.0) was used for single nucleotide polymorphisms/variants (SNPs/SNVs) and short insertions/deletions (INDELs) (<50 bp) calling. Samtools (version 1.22.1) and Picard software (version 2.22.1) packages were used to generate high-quality binary alignment map (BAM) data by removing duplicate reads. Variants were annotated for analysis using the single nucleotide polymorphism database (dbSNP, version 5.3, https://www.ncbi.nlm.nih.gov/snp/), gnomAD exomes database (version 4.1.0, https://gnomad.broadinstitute.org/) and Chigene in-house minor allele frequency (MAF) database. Tools of pathogenicity prediction like REVEL (Rare Exome Variant Ensemble Learner) and AlphaMissense were used for predicting possible impact of variants. As a prioritized pathogenicity annotation to the American College of Medical Genetics and Genomics (ACMG) guidelines, Online Mendelian Inheritance in Man (OMIM, https://www.omim.org/), Human Gene Mutation Database (HGMD, https://www.hgmd.cf.ac.uk/) and ClinVar databases (https://www.ncbi.nlm.nih.gov/clinvar/) were used as conferences of pathogenicity of every variant.

### Variants classification

As per the guidelines of ACMG for interpreting sequence variants, variants were classified. Classification considered the position of the variant in the human genome, MAF, the pathogenicity prediction of variants, disease mechanism, clinical phenotypes, literature evidence, evolutionary conservation.

### Variants verification

Fetal and parental gDNA samples was used for the verification of this variant. All reactions were carried out in a total volume of 20 μL using the Taq DNA polymerase. After confirmation of the size of the amplicons, the PCR products were purified by standard protocol. The reaction system was: 2 μL DNA (10 ng/μL), 1 μL forward primer, 1 μL reverse primer, 10 μL PCR Master Mix, 6 μL free RNA H_2_O. The following protocol of PCR: initial denaturation at 95 °C for 3 min, followed by 30 cycles at 95 °C for 30 s, 60 °C for 30 s, and 72 °C for 30 s, with a final elongation at 72 °C for 10 min. The PCR products were electrophoresed on a 2.0% agarose gel and were sequenced. Sanger sequenced using the BigDye Terminator v3.1 Cycle Sequencing Kit (ABI 3730 Applied Biosystem, USA) for the verification of candidate variants. The identification of this variant was performed using Chromas software (version 2.2.6, Technelysium, Australia).

### Construction of the *SLC6A9* variants *in vitro* and transfection

Two mutant *SLC6A9* expression vectors pCMV-Flag-Neo(EGFP)-*SLC6A9*-m1/m2 (*SLC6A9*-m1/m2), one wild-type pCMV-Flag-Neo(EGFP)-*SLC6A9*-wt (*SLC6A9*-wt) and one empty vector negative control mammalian expression vectors were constructed and purchased from Bioeagle Biotech Company Ltd (Wuhan, China). Human embryonic kidney (HEK)-293T cells were transfected with the above four expression vectors in separate tubes. The constructed expression vectors were verified using Sanger sequencing. Then, 6 h after the transfection, we gently removed the lipofectamine-containing medium, replaced the medium with Dulbecco's modified Eagle medium (DMEM) containing 5% fetal bovine serum (FBS), and incubated the slide at 37℃ in a 5% CO_2_ incubator until day 2. On day 3, the samples were collected and used in a reverse transcription-polymerase chain reaction (RT-PCR) to quantify the *SLC6A9* mRNA expression and the Western blot (WB) assay to quantify the SLC6A9 protein expression. Each cell line underwent three independent replicates of the RT-PCR and WB assays. The RT-PCR and Western blotting experiments were conducted in accordance with the manufacturer's instructions.

### Reverse transcription-quantitative polymerase chain reaction (RT-qPCR)

Wild-type (*SLC6A9*-wt), mutant (*SLC6A9*-m1/m2) and negative control expression vectors were respectively transfected into HEK293T cells. All samples were collected at 48 h post-transfection for subsequent analysis. The transfected cells were collected by centrifugation, followed by total RNA extraction using the Trizol method. Total RNA concentration and quality were checked using a NanoDrop 2000 spectrophotometer (Thermo Fisher Scientific, USA). Complementary DNA (cDNA) was synthesized using NovoScript® Plus All-in-one 1st Strand cDNA Synthesis SuperMix (Novoprotein, China) following the manufacturer's instructions. The cDNA concentration was about 1,200 ng/μL. The total reaction system was 20 µL, with 500 ng of total RNA used as a template. Quantitative PCR was performed using the Maxima SYBR Green/ROX qPCR Master Mix (2×) (Thermo Fisher Scientific, USA). Relative *SLC6A9* mRNA expression levels were determined using 2^−ΔΔCt^ method, and the *ACTB* gene was used as the reference gene. The experimental values were normalized to the empty-48 h condition, designated as the internal control. The qRT-PCR reaction was performed in triplicate, and mean values and standard deviations were calculated from the obtained data.

### Western blot (WB)

Wild-type (*SLC6A9*-wt), mutant (*SLC6A9*-m1/m2) and negative control expression vectors were separately transfected into HEK293T cells. All samples were collected at 48 h post-transfection for subsequent analysis. Western blot analysis was subsequently performed using standard protocol. Equal amounts of protein were resolved by sodium dodecyl sulfate-polyacrylamide gel electrophoresis (SDS-PAGE) and subsequently transferred onto polyvinylidene fluoride (PVDF) membranes. Membranes were blocked with 5% non-fat milk in a tris buffered saline with tween-20 (TBST) solution for 2 h at room temperature. Anti-Flag (MBL, Japan, dilution ratio is 1:1000) and anti-GAPDH dilutions were added respectively and incubated at 4 °C overnight. Horseradish peroxidase (HRP)-labeled secondary antibodies were added and incubated at room temperature for 1 h. GAPDH served as the loading control and internal reference protein. The loading amount of WB protein was 10 μg/well. Blot bands were quantified by densitometry using ImageJ software (ImageJ2). Protein levels were normalized to GAPDH. The relative SLC6A9 protein expression levels are quantified by the fold change (FC) in the ratio of the grayscale between the SLC6A9 protein band and the internal reference protein band.

### Statistical analysis

Multiple comparisons across these experimental groups were performed using the Holm-Šídák multiple comparisons test in Prism software (GraphPad Prism 10.6.0). *P* < 0.05 was considered statistically significant.

### Literature review

We systematically searched the PubMed database for relevant published studies on the research topic using “*SLC6A9* gene” as keywords. The search time was from the establishment of the databases to June 30, 2026. We reviewed *SLC6A9*-related cases with availability of clinical data including relevant genetic testing results, prenatal/postnatal manifestations.

## Results

### Genetic analysis and confirmation

Trio-WES identified novel compound heterozygous missense variants c.859C > T (p.Arg287Trp) and c.1120T > C (p.Cys374Arg) in the *SLC6A9* gene (NM_201649.4) These variants were further verified by Sanger sequencing, confirming that the heterozygous variant c.859C > T (p.Arg287Trp) was inherited from the mother and the heterozygous variant c.1120T > C (p.Cys374Arg) was inherited from the father (Supplementary Figure 1). ① m1 (c.859C > T): The allele frequency of the heterozygous variant c.859C > T was 0.000002543 in the gnomAD (v4.1.1). However, it was not observed in East Asian populations, nor were homozygous carriers observed. The score of REVEL for this variant, an ensemble method for predicting the pathogenicity of missense variants based on a combination of scores from 13 individual tools, was 0.71. ② m2 (c.1120T > C): The allele frequency of the heterozygous variant c.1120T > C was 0 in the gnomAD (v4.1.1). The score of REVEL for this variant was 0.73. As per the interpretation guidelines by ACMG (4), both variants were classified as variants of uncertain significance (VUS) (PM2_Supporting + PP3_Supporting). We have submitted these variants to a public database [Leiden Open Variation Database (LOVD)], and the accession number can be found at the following URL: https://databases.lovd.nl/shared/individuals/00484148.

### Functional analysis of the missense variants

Sanger sequencing was performed to validate the full-length *SLC6A9* gene cDNA in both wild-type and mutant vectors. Sanger sequencing confirmed that the wild-type and mutant expression vectors were successfully constructed (Supplementary Figure 2). To evaluate the effect of these *SLC6A9* variants, our functional studies revealed that these *SLC6A9* variants didn't markedly decreased mRNA expression of *SLC6A9* gene (Figure 2A), but leading to a reduction of mutant SLC6A9 protein (Figure 2B) compared to the *SLC6A9*-wt groups. The mean values and *P*-values of qPCR and WB are presented in Supplementary Table. These results were consistent with the findings of Wang et al. (5) (e.g., variants p.Y206F, p.F207Y, p.R333H, p.E338K, p.E446K, p.R643H, p.R662W).

**Figure 2:**
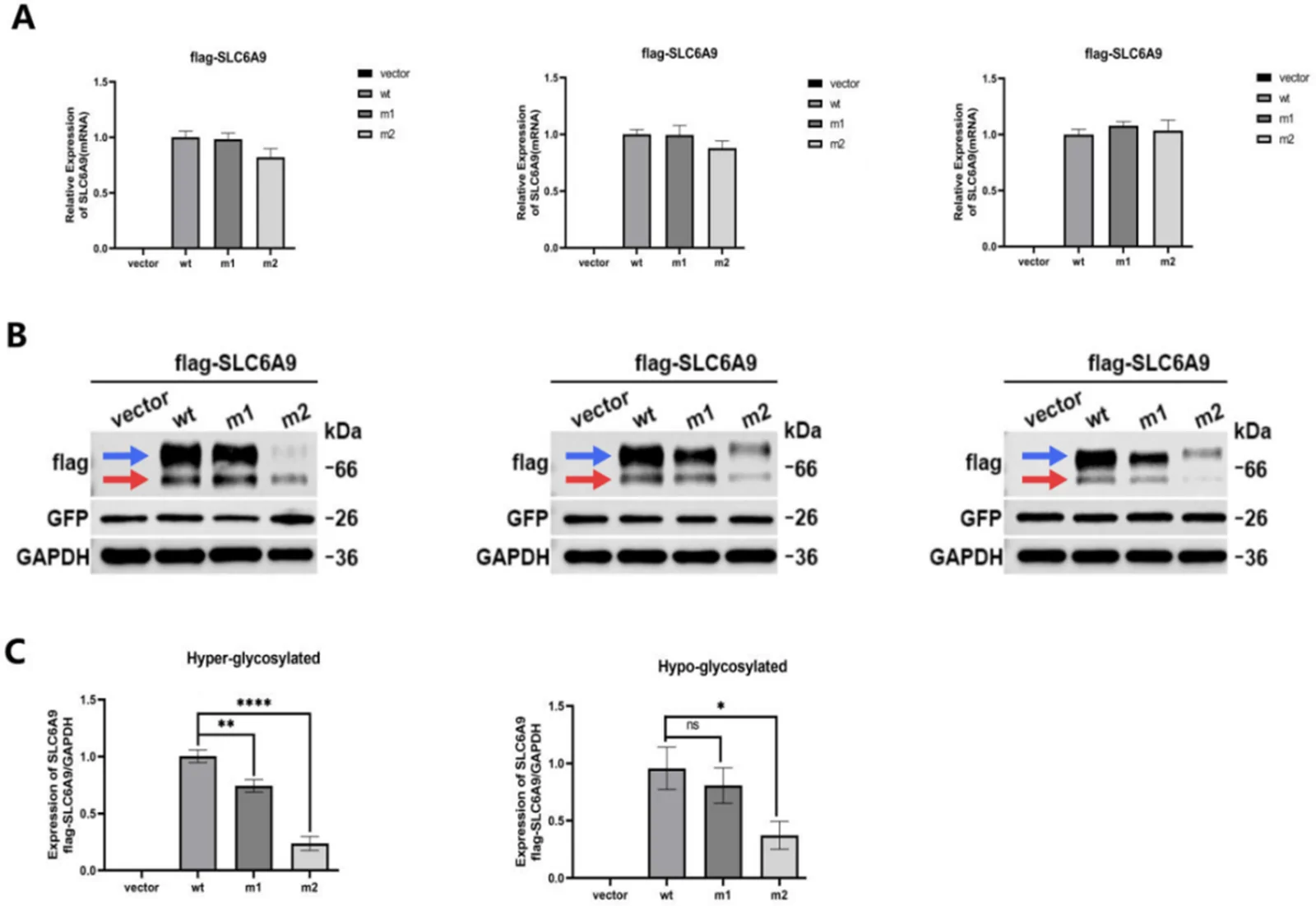
The relative *SLC6A9* mRNA and SLC6A9 protein expression. **(A)** Relative mRNA expression levels of the *SLC6A9* gene: The experimental values were normalized to the wt-48 h condition designated as the internal control. The results of RT-PCR analysis showed that the mRNA expression of mutant *SLC6A9* cell lines was not significantly decreased. Each cell line was subjected to three independent replicates of the RT-PCR assay. The difference between *SLC6A9*-wt and *SLC6A9*-mut was assessed using the Holm-Šídák test. **(B)** Western blot analysis of SLC6A9 protein expression: Western blot analysis revealed two bands neared the 60 and 80 kDa positions in both the wild-type and the mutant groups; the width and color of these bands in the wild-type groups appeared to be lower than those in the mutant groups. The blue arrow indicated the hyper-glycosylated SLC6A9 protein (approximately 80 kDa). The red arrow denoted the hypo-glycosylated intermediate SLC6A9 protein (approximately 60 kDa). **(C)** Quantification of SLC6A9 protein expression levels: The relative SLC6A9 protein expression was quantified by the fold change (FC). No statistically significant difference was observed in the expression level of the hypo-glycosylated SLC6A9 protein between the *SLC6A9*-m1 groups and the wild-type SLC6A9-wt groups, but the expression levels of the hypo-glycosylated SLC6A9 protein were significantly decreased in SLC6A9-m2 groups. The expression levels of the hyper-glycosylated SLC6A9 protein were significantly decreased in *SLC6A9*-m1/m2 groups. The difference between *SLC6A9*-wt and *SLC6A9*-m1/m2 was evaluated by Holm-Šídák test (*P < 0.05, **P < 0.01, ****P < 0.0001, ns, not significant).

## Discussion

Solute carriers (SLC) are a superfamily of transporter proteins that play a crucial role in the transport of substances (such as ions, amino acids, carbohydrates, lipids, nucleotides, vitamins, and drugs) across cellular membranes. They are involved in various physiological functions, including nutrient uptake, metabolic regulation, and waste expulsion (6). The SLC6A9 protein is a member of the SLC transporter protein superfamily. The *SLC6A9* gene (solute carrier family 6 member 9, OMIM*601019) is located on chromosome 1p34.1. The transcript of *SLC6A9* gene (NM_201649.4) has 14 exons with the transcript length of 2,330 base pairs. Its translation product consists of 706 amino acid residues. The precursor SLC6A9 protein undergoes preliminary partial N-glycosylation modifications (hypo-glycosylated) in the endoplasmic reticulum to form an intermediate product of approximately 60 kDa. This hypo-glycosylated SLC6A9 protein is further processed by the Golgi apparatus (with addition of complex oligosaccharide chains) to yield the fully glycosylated (hyper-glycosylated) mature membrane glycine transporter 1 (GlyT1), weighing about 80 kDa (7). The mature GlyT1 is trafficked and localized to the plasma membrane, it exhibits full substrate binding and transport activity. GlyT1 is widely distributed in the central nervous system and peripheral organs. In neurons (both excitatory and inhibitory) and glial cells (astrocytes, oligodendrocytes, and ependymal cells), the sodium- and chloride-dependent GlyT1 clears glycine from the synaptic cleft, regulates inhibitory glycine signaling in the central nervous system, and maintains dynamic glycine homeostasis (8).

From 2003 to 2005, several researchers successively reported findings on *Slc6a9* gene knockout animal models. Homozygous *Slc6a9* knockout mice exhibited disrupted respiratory rhythms, severe respiratory distress, and generalized hypotonia after birth, ultimately dying within 12 h of birth (9); heterozygous *Slc6a9* knockout mice survived and exhibited normal phenotypes in adulthood, showing no difference from wild-type mice (10). The s*lc6a9* gene mutant zebrafish displayed motor behavior deficits during the first 2 days of development, such as diminished escape responses upon tactile stimulation and the absence of swimming (11). It was not until 2016 that Majid Alfadhel et al. (3) and Kurolap et al. (12) reported six documented cases of three homozygous human *SLC6A9* gene variants associated with neonatal respiratory distress, hypotonia that rapidly transformed into hypertonicity with pronounced startle-like clonus jerks, arthrogryposis multiplex congenita, congenital talipes equinovarus, elevated CSF glycine concentrations with or without elevated plasma glycine concentrations, and neonatal lethality.

After a literature review, we found that a total of 14 documented cases with *SLC6A9*-related glycine encephalopathy from 7 pedigrees have been reported in six literature to date. There were four homozygous missense and three homozygous nonsense *SLC6A9* variants in these 14 cases (Table 1). The case reported in this study was the first genetically confirmed case with compound heterozygous missense *SLC6A9* variants. Consanguineous marriages were identified in six families. Five of 14 cases were fetuses who underwent pregnancy termination, and the remaining nine were live-born infants, of whom five were delivered preterm (before 35 weeks' gestation). The specific phenotype of *SLC6A9*-related glycine encephalopathy was characterized by elevated CSF glycine concentrations alongside normal or elevated serum glycine concentrations. Common clinical manifestations included: death due to respiratory failure within 7 months after birth (78%, 7/9), a phenotypic transition from hypotonia to hypertonia, startle-clonic seizures, and epileptiform episodes within 1–2 weeks after birth (67%, 6/9), cranial and facial dysmorphism (89%, 8/9, such as microcephaly, macrocephaly, trigonocephaly, broad forehead, esotropia, thick or sparse eyebrow, long eyelashes, wide-set eyes, hypertelorism, cleft palate, low-set ears, posterior rotated ears retrognathia, deep philtrum, anteverted nostrils, collapsed nasal bridge, ptosis), arthrogryposis (67%, 6/9), congenital talipes equinovarus (56%, 5/9); as well as non-specific phenotypes such as other limb or femoral deformities (e.g., high-arched feet, thoracic kyphosis, joint dislocations/contactures, scoliosis, knee hyperextension, overlapping fingers/toes, absence of patellae, decreased palmar wrinkling, single palmar creases). Additional abnormal phenotypes may include cryptorchidism, bilateral hydrocele, micropenis, inguinal hernia, atrial septal defect, hypertension, hypotension, hearing impairment, hirsutism, motor and sensory polyneuropathy, abnormal visual evoked responses, and cutaneous hyperpigmentation. We found that no significant differences in clinical phenotypes were observed between individuals harboring missense *SLC6A9* variants and those with nonsense *SLC6A9* variants. The penetrance of missense/nonsense in the *SLC6A9* gene appeared to be complete. All 12 cases with available prenatal ultrasound records exhibited nonspecific prenatal phenotypic features. The most frequently observed abnormalities were increased NT (6/12, 50%), polyhydramnios (5/12, 42%), talipes equinovarus (6/12, 50%), and arthrogryposis multiplex congenita (5/12, 42%). This newborn reported by us was the first documented neonatal fatality linked to the compound heterozygous missense *SLC6A9* variants in China. In addition to the aforementioned clinical findings (recurrent apneic episodes, respiratory distress after birth, cranial and facial dysmorphism, a phenotypic transition from hypotonia to hypertonia, talipes equinovarus), this neonatal case also presented with necrotizing enterocolitis, pneumonia, anemia (not previously described in the literature), and died from respiratory failure on the fourth day after birth. This family had a documented history of one adverse pregnancy outcome and the postpartum clinical presentations closely matched the clinical phenotypes of *SLC6A9*-related glycine encephalopathy in the literature. Fetal talipes equinovarus was observed in two pregnancies within this family, suggesting that this phenotype may represent one of the characteristic manifestations of *SLC6A9*-related glycine encephalopathy during the prenatal stage and warranted clinical attention.

**Table 1:** Summary of clinical phenotypes in patients with previously reported *SLC6A9* gene variants.

*SLC6A9* variants (NM_201649.4)	Pedigree and case number	Prenatal manifestations	Main manifestations after birth	Glycine concentration	Brain MRI	Reference
c.1219A > G (p.Ser407Gly) homozygosity, heterozygous parents, consanguineous marriage	Pedigree 1 case 1	Polyhydramnios	Female, 15-month-old, full term birth, recurrent apneic episodes, respiratory distress after birth, hypotonia that rapidly transformed into hypertonicity with pronounced startle-like clonus jerks, cranial and facial dysmorphism, **talipes equinovarus**, global delay	Elevated normal serum glycine level (288 μmol/L)↑	Bilateral subinsular white matter T2 hyperintensity, right head of caudate atrophy with tiny cyst, and bilateral scattered subcortical and periventricular white matter changes	(3)
				Elevated CSF glycine level (21 μmol/L)↑		
c.928_932del (p.Lys310PhefsTer31) homozygosity, heterozygous parents, consanguineous marriage	Pedigree 2 case 2	NT4.2 mm, fourth ventricle dilation, bilateral clubfeet, polyhydramnios	Female, 2-year-old, born at 33 weeks of gestation, recurrent apneic episodes, respiratory distress after birth, hypotonia that rapidly transformed into hypertonicity with pronounced startle-like clonus jerks, cranial and facial dysmorphism, arthrogryposis, **talipes equinovarus**, other limb or skeletal deformities, hypertension	Normal serum glycine level	Enlargement of lateral and third ventricles, mildly excessive extra-axial fluid, a focal area of restriction diffusion in the left hemisphere of the cerebellum	(12)
				Elevated CSF glycine level (22 μmol/L) ↑		
	Pedigree 2 case 3	NT6.0 mm, hydrops (edema of the abdominal wall and scalp)	termination of pregnancy	NA	NA	
c.1717C > T (p.Gln573*) homozygosity, heterozygous parents, consanguineous marriage	Pedigree 3 case 4	NA	Female, born at 35 weeks of gestation, respiratory distress after birth, hypotonia that rapidly transformed into hypertonicity with pronounced startle-like clonus jerks, cranial and facial dysmorphism, arthrogryposis, other limb or skeletal deformities, **death due to recurrent apnea episodes on the 18th day after birth**	Elevated CSF glycine level ↑	NA	
	Pedigree 3 case 5	Arthrogryposis	Female, born at 41 weeks of gestation, respiratory distress after birth, hypotonia that rapidly transformed into hypertonicity with pronounced startle-like clonus jerks, cranial and facial dysmorphism, arthrogryposis, other limb or skeletal deformities, **death due to respiratory failure and bradycardia on the second day after birth**	NA	NA	
	Pedigree 3 case 6	Arthrogryposis, hyperextension of knees, bilateral club feet, no movement of feet or elbows, deviations of his fingers	Male, born at 30 weeks of gestation, respiratory distress after birth, hypotonia that rapidly transformed into hypertonicity with pronounced startle-like clonus jerks, cranial and facial dysmorphism, arthrogryposis, talipes equinovarus, other limb or skeletal deformities, cryptorchidism, inguinal hernia, atrial septal defect, hypotension, conductive hearing impairment, **death due to respiratory failure on the 7th month after birth**	Normal serum glycine level	Thin corpus callosum and bilateral optic nerve atrophy	
				elevated CSF glycine level (25–31 μmol/L) ↑		
c.352G > A (p.Val118Met) homozygosity, heterozygous parents, non-consanguineous marriage	Pedigree 4 case 7	Increased NT, arthrogryposis	Termination of pregnancy	NA	NA	(13)
	Pedigree 4 case 8	Increased NT, arthrogryposis	Termination of pregnancy	NA	NA	
c.997delC (p.Arg333Alafs*3) homozygosity, heterozygous parents, consanguineous marriage	Pedigree 5 case 9	Polyhydramnios, cryptorchidism, bilateral clubfeet, lack of lower limb movements	Male, born at 38 weeks of gestation, respiratory distress after birth, hypotonia that rapidly transformed into hypertonicity with pronounced startle-like clonus jerks, cranial and facial dysmorphism, arthrogryposis, talipes equinovarus, other limb or skeletal deformities, scoliosis, micropenis, cryptorchidism, death **due to respiratory failure on the 13th day after birth**	Normal serum glycine level (234.9 μmol/L)	Normal	(14)
				Elevated CSF glycine level (49.5 μmol/L) ↑		
	Pedigree 5 case 10	NT6.2 mm, bilateral clubfeet	natural miscarriage at 16 weeks	NA	NA	
c.469G > A (p.Glu157Lys) homozygosity, heterozygous parents, consanguineous marriage	Pedigree 6 case 11	NT2.5 mm, macrocephaly, polyhydramnios, hydrops, absent fetal movement	Male, born at 32 weeks of gestation of gestation, **death due to bradycardia and hydrops within the first postnatal hour**, cranial and facial dysmorphism, thoracic kyphosis, hypertrichosis, arthrogryposis, talipes equinovarus, other limb or skeletal deformities, hemosiderosis, lung hypoplasia	NA	NA	(15)
	Pedigree 6 case 12	Polyhydramnios, bilateral clubfeet, knee hypextension, hydrops	Female, born at 32 weeks of gestation, death **due to hydrops within the first postnatal hour**, cranial and facial dysmorphism, arthrogryposis, talipes equinovarus, other limb or skeletal deformities, hypertrichosis, short neck, thoracic kyphosis, atrial septal defect, hemosiderosis	NA	NA	
	Pedigree 6 case 13	Bilateral clubfeet, bilateral flexion contractures of elbows, IUGR	Terminated at 16 weeks of gestation, due to absent fetal heart activity, cord hyper coiling, hemosiderosis	NA	NA	
c.454C > T (p.Pro152Ser) homozygosity, heterozygous parents, consanguineous marriage	Pedigree 7 case 14	NA	Female, born at term, poor sensorium after birth, hypotonia, seizures, **death due to catecholamineresistant shock and cardiorespiratory failure at 72 h of life**	Normal serum glycine level (519 μmol/L)	Mild cerebral edema	(16)
				Elevated CSF glycine level (62.61 μmol/L) ↑		
c.859C > T(p.Arg287Trp), c.1120T > C(p.Cys374Arg) heterozygous parents, non-consanguineous marriage	Pedigree 8 case 15	Bilateral clubfeet, bilateral choroid plexus cysts	Female, born at 39 weeks of gestation, dyspnea and recurrent apnea after birth, hypotonia that rapidly transformed into hypertonicity, cranial and facial dysmorphism, arthrogryposis, talipes equinovarus, other limb or skeletal deformities, necrotizing enterocolitis, pneumonia, anemia, **death due to respiratory failure on the 4th day after birth**	Normal serum glycine level (699.00 μmol/L)	NA	This case

IUGR, intrauterine growth retardation; NT, nuchal translucency; MRI, magnetic resonance image; CSF, cerebrospinal fluid; NA, not available.

Based on three cases with *SLC6A9* nonsense variants and *Slc6a9* gene knockout animal models, we postulated that the pathogenic mechanism of *SLC6A9*-related glycine encephalopathy may be associated with the loss-of-function (LoF) effect of *SLC6A9* variants. The gene dosage sensitivity curation from Clinical Genome Resource (ClinGen) database does not record the score of haploinsufficiency of *SLC6A9* gene. The “gene2phenotype” database records that the pathogenic mechanisms of *SLC6A9* gene variants may be associated with the loss-of-function (last updated: 2025-01-28). To validate the hypothesis proposed in this study, we conducted *in vitro* cellular function assays on two identified missense variants in this case. By constructing two mutant *SLC6A9* expression vectors and transfecting them into human HEK293T cells, we measured the mRNA and protein expression levels of the *SLC6A9* gene. This study results demonstrated no significant difference in mRNA expression levels between the two mutant groups compared to the wild-type group, but a marked reduction in SLC6A9 protein expression was observed. These findings suggested that these two missense variants may impair SLC6A9 protein expression, compromising glycine transport efficiency and activity. This finding aligned with the hypothesis of a “LoF pathogenic mechanism” for *SLC6A9* gene variants.

Currently, direct functional evidence for the pathogenicity of *SLC6A9* LoF variants remains extremely limited, and the number of well-documented cases is also limited. Nonetheless, emerging experimental evidence suggests that certain *SLC6A9* variants may indeed exert the LoF effects. This review synthesizes current knowledge on the potential pathogenic mechanisms underlying *SLC6A9* LoF variants, aiming to establish a coherent theoretical foundation, and guide future functional validation studies and classification of *SLC6A9* variants. ① For *SLC6A9* nonsense variants (13): Nonsense variants introduced a premature termination codon (PTC), causing the nonsense-mediated mRNA decay (NMD). Truncated proteins exhibited precluded normal N-glycosylation, thereby compromising structural stability and rendering them susceptible to rapid degradation by the intracellular ubiquitin-proteasome system (e.g., p.Lys310Phefs*31); some truncated proteins may undergo incomplete N-glycosylation and subsequently form dimers or oligomers, leading to their retention in the endoplasmic reticulum and failing to traffic to the cellular membrane (e.g., p.Gln573*); owing to the loss of essential structural or functional domains, some truncated variants may completely lack glycine transport activity. Nonsense variants resulted in the loss of GlyT1 function via the aforementioned molecular mechanism. ② For *SLC6A9* missense variants: missense variants (e.g., p.Val118Met, p.Ser407Gly) did not alter SLC6A9 protein expression levels, post-translational modification patterns, or subcellular localization to the plasma membrane; however, they significantly diminished GlyT1 substrate binding affinity and transport efficiency, thereby compromising glycine uptake capacity and leading to a functional loss of GlyT1 activity (13). Our study elucidated an alternative pathogenic mechanism underlying missense *SLC6A9* variants. Specifically, these variants do not alter *SLC6A9* mRNA expression levels but lead to a significant reduction in SLC6A9 protein expression (including both fully and partially N-glycosylated forms). This phenomenon was similar to prior observations reported by Wang et al. (5) (e.g., p.Y206F, p.F207Y, p.R333H, p.E338K, p.E446K, p.R643H, p.R662W). The underlying pathogenic mechanism is proposed as follows: missense variants may reduce the stability of SLC6A9 protein (e.g., by impairing its N-glycosylation modification or three-dimensional structure), thereby activating intracellular proteasome and lysosome degradation pathways, which decreased the total amount of functional GlyT1 on the cellular membrane (12); missense variants may form heterodimers or polymeric complexes with wild-type GlyT1, which couldn't be transported to the cellular membrane via the endoplasmic reticulum-Golgi trafficking pathway but instead accumulated and were degraded intracellularly, leading to a significant reduction in functional GlyT1 levels on the cellular membrane (17). In summary, *SLC6A9* gene variants may impair the quantity or function of GlyT1 on the cellular membrane via multiple molecular mechanisms, including reduced structural stability, disruption of essential structural domains, aberrant post-translational modifications, and diminished substrate affinity or transport efficiency, thereby exerting a loss-of-function effect. Nevertheless, this study is subject to several limitations. First, the study is limited by a small sample size, as the compound heterozygous variants were identified in only a single family; therefore, the pathogenicity of these variants and phenotypic spectrum warrant further validation through additional familial and cohort-based investigations. Second, *in vitro* cellular assays inherently lack the physiological complexity of the *in vivo* environment; consequently, the functional consequences of the missense *SLC6A9* variants require corroboration in more physiologically relevant models, such as animal models or patient-derived induced pluripotent stem cells (iPSC). Third, the rapid clinical deterioration observed in the affected proband precluded timely collection of CSF, thereby limiting the assessment of CSF glycine concentrations. Ultimately, owing to funding constraints, experimental evidence supporting a LoF mechanism underlying these *SLC6A9* missense variants remains incomplete. Complementary functional investigations into these *SLC6A9* missense variants still necessitate additional experimental validation, including N-glycosylation analysis, subcellular localization assays, and electrophysiological characterization of glycine-induced GlyT1.

In conclusion, this study identifies and functionally validates novel compound heterozygous missense variants in the *SLC6A9* gene through comprehensive analysis of a family with adverse obstetric outcomes. To our knowledge, We presented the first reported neonatal lethality caused by compound heterozygous missense variants in the *SLC6A9* gene and comprehensively delineated the prenatal and postnatal phenotypic spectrum associated with *SLC6A9*-related glycine encephalopathy, thereby offering novel molecular insights to support prenatal diagnosis and informed genetic counseling. Also, this study integrated *in vitro* cellular functional assays with evidence from published case reports, *SLC6A9* gene knockout animal models, and independent cellular functional studies reported by other research groups, thereby providing preliminary mechanistic evidence that the pathogenic mechanism underlying *SLC6A9*-associated glycine encephalopathy may be attributable to the LoF effect conferred by pathogenic variants in the *SLC6A9* gene.

## Data Availability

The original contributions presented in the study are publicly available. This data can be found here: The data presented in the study are deposited in Quark repository: https://pan.quark.cn/s/5ad221c28c12, accession 5ad221c28c12.
