# Supplementary material for: Case Report: Neonatal lethality caused by novel compound heterozygous missense variants of SLC6A9 gene
Source: Front Pediatr. 2026 Sep 25;14:1945449. doi: 10.3389/fped.2026.1945449 (PMC13649721; doi:10.3389/fped.2026.1945449)
Supplement: Supplementary file 1 [file Supplementaryfile1.docx]

**Supplementary Figure 1:** Sanger sequencing of compound heterozygous missense variants

Sanger sequencing confirmed that the newborn carried the heterozygous variant c.859C>T (p.Arg287Trp) inherited from the mother and the heterozygous variant c.1120T>C (p.Cys374Arg) inherited from the father. The blue box marked the positions of the variants.

**Supplementary Figure 2**: Sanger sequencing of mutant expression plasmids

Sanger sequencing confirmed that the mutant expression plasmids were successfully constructed. The red box indicated the variants.
